# Enzymatic and Antimicrobial Activity of Biologically Active Samples from *Aloe arborescens* and *Aloe barbadensis*

**DOI:** 10.3390/biology10080765

**Published:** 2021-08-11

**Authors:** Maja Leitgeb, Kaja Kupnik, Željko Knez, Mateja Primožič

**Affiliations:** 1 Laboratory for Separation Processes and Product Design, Faculty of Chemistry and Chemical Engineering, University of Maribor, Smetanova ulica 17, SI-2000 Maribor, Slovenia; kaja.kupnik@um.si (K.K.); zeljko.knez@um.si (Ž.K.); mateja.primozic@um.si (M.P.); 2 Faculty of Medicine, University of Maribor, Taborska ulica 8, SI-2000 Maribor, Slovenia; 3 Faculty of Mechanical Engineering, University of Maribor, Smetanova ulica 17, SI-2000 Maribor, Slovenia

**Keywords:** *Aloe arborescens*, *Aloe barbadensis*, antimicrobial activity, bioactivity, enzymes

## Abstract

**Simple Summary:**

Antimicrobial resistance is one of the major threats to public health, and additional concerns are reduced efficacy and increased toxicity of synthetically derived drugs. Hence, it is all the more important to research new antimicrobials derived from natural sources. *Aloe* spp. have long been acknowledged in traditional medicine, as their ability of treating skin and digestive problems, wound healing, anti-inflammatory, antimicrobial and other promising properties are known. This study presents the content of various bioactive substances in samples of two *Aloe* spp., *Aloe arborescens* and *Aloe barbadensis*, and their enzymatic, antioxidant and antimicrobial activity. Obtained bioactive compounds with antimicrobial effect have a huge potential to inhibit the growth of microorganisms that are extremely susceptible to gaining resistance and could be used in versatile applications in the cosmetics, food, medical and pharmaceutical industries.

**Abstract:**

Recently, the use of *Aloe* species has become very widespread. These are extensively used as a nutraceutical in a variety of health care products and food supplements. In addition, the occurrence of the quickly adaptable microorganisms, particularly bacteria, which can develop resistance to antibiotics, is a major problem for public health, and therefore, it is necessary to search for new antimicrobials. In our study, the content of total phenols, proanthocyanidins, and proteins in fresh and lyophilized samples of *A. arborescens* and *A. barbadensis* and their ethanol extracts was investigated. Furthermore, enzymatic and antioxidant activity of samples were studied. Since antimicrobial activity of fresh samples was determined in our latest research, a more detailed study of antimicrobial effectiveness of *A. arborescens* and *A. barbadensis* (lyophilized, extracts) was performed. Ethanol extracts in particular contain higher concentrations of bioactive substances and show the topmost antioxidant activity. The novelty of the study refers to the observation of industrially important enzyme activities such as α-amylase, cellulase, lipase, peroxidase, protease, and transglutaminase in the samples as well as the microbial growth inhibition rates determination (MGIR) at different concentrations of added aloe samples. All samples inhibited the growth of all tested microbial cells. MIC_90_ for *A. arborescens* and *A. barbadensis* were also determined in case of *B. cereus*, *P. aeruginosa*, *P. fluorescens,* and *S. aureus*. The results of our study tend to give credence to the popular use of both aloes in medicine and in the cosmetic, food, and pharmaceutical industries.

## 1. Introduction

Much of today’s biomass on planet Earth is represented by microbes. Although they are not perceived by the human eye, they are extremely influential in people’s lives. Among other things, many microbes constitute the natural microbial flora in the human body. Microbes are important for the functioning of the human body because they participate in the food chain and other important processes [1,2]. The problem are pathogenic microorganisms. These are dangerous to humans and can cause diseases. Diseases caused by pathogens are a major problem for human health. Antibiotics that treat bacterial infections are no longer so effective because of microorganisms’ property of being able to develop drug resistance. Especially bacteria adapt quickly and therefore negate the effects of drugs [3,4,5]. 

**Figure 1:**
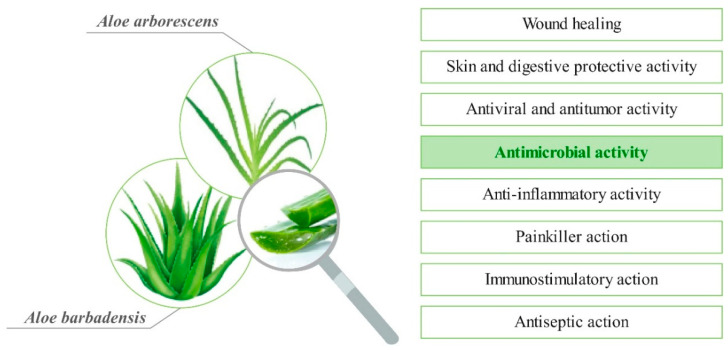
Medical benefits of *A. arborescens* and *A. barbadensis* [10,11,12,13,14,15].

Because drugs can have a toxic effect on humans, there is a growing interest in alternative, natural antimicrobial agents that would inhibit the growth and reproduction of opportunistic bacteria. Plants and natural preparations that have potential to inhibit the growth of microorganisms and ensure lower toxicity than drugs are nowadays very interesting [6]. Among these plants are also *Aloe arborescens* and *Aloe barbadensis. A. barbadensis* is well known as *Aloe vera* [7], while generally *A. arborescens* is less known. Although most people are unfamiliar with it, several countries have been using *A. arborescens* for many years in traditional medicine [8]. Both have many beneficial health effects (Figure 1) and can be used for different purposes [9]. Sánchez et al. [10] recently published a review on the updated pharmacological properties of *A. barbadensis* and its main active ingredients. 

Most commonly, both aloes are used to treat skin problems, abdominal problems, digestive problems, for the healing of wounds and burns, and as an anti-inflammatory and antimicrobial agent [16,17]. Because aloes were thus used in traditional medicine, the food industry took advantage of this and began using aloe extracts in food and dietary supplements as a functional food [18,19]. Lately, the aloe-derived gel has become increasingly popular. In most cases, it is used in the cosmetics industry and the pharmaceutical industry and as a nutraceutical in the food industry [16,20,21]. 

*A. barbadensis* contains mostly water and approx. 0.7% is represented by various bioactive substances, of which 75 are known [22,23]. These include nutritive constituents as carbohydrates (polysaccharides called glucomannans, etc.), vitamins (ascorbic acid, carotenoids, tocopherols, vitamin B1, B2, B6, niacin, folic acid, etc.), enzymes (cellulase, carboxypeptidase, amylase, bradykinase, oxidase, and catalase, etc.), minerals (magnesium, calcium, iron, copper, zinc, and chromium, etc.), proteins (glycoproteins), and amino acids (seven essential amino acids and additionally 20 out of 22 amino acids that occur naturally). Nonnutritive constituents include phenolic compounds (e.g., anthraquinones), organic acids (salicylic, lactic, acetic, malic, and succinic acid, etc.), and phytosterols (lupeol, cholesterol, β-sitosterol, campesterol, etc.) [24,25,26]. Certain molecules in aloe have antibacterial, antiviral, and antifungal properties. Above all, *A. vera* is able to slow down the development of bacteria and fungi [27] due to the presence of two organic acids, cinnamic and chrysophanic acid also known as chrysophanol [28]. The anthraquinone group also includes aloin, oleic acid, and aloe-emodin, which have proven painkiller and antimicrobial effects [22,29,30,31,32]. 

The gel content per leaf is lower in *A. arborescens* than in *A. barbadensis* [28], which makes it less attractive for commercial purposes; nevertheless, it is interesting because of its potential medicinal properties. Different studies indicate a good inhibitory effect of *A. barbadensis* on some opportunistic bacteria and fungi, including *Staphylococcus aureus* [16,22,33,34,35,36,37,38,39], *Escherichia coli* [3,22,35,38,39,40], *Pseudomonas aeruginosa* [16,22,35,36,37,38,39,40,41], *Pseudomonas fluorescens* [3], *Bacillus cereus* [34], and *Candida albicans* [37,40], which were also tested in presented study. 

“Medicinal aloe” is another name for *A. arborescens* as it has been used as a folk medicine in many countries around the world for many years [42]. *A. arborescens* has great potential, but not much research in this field has been done. Our published article [1] based on the study and comparison of the inhibitory effect of natural gel and juice of *A. arborescens* and *A. barbadensis* brings a very important contribution to this field. As the exceptional antimicrobial efficacy of all-natural gels and juices from both aloes was found, we continued with research in this area. Therefore, the focus of our presented research was to study the content of various bioactive secondary metabolites that could also contribute to the inhibitory effect of these aloe species and to further study antimicrobial activity of *A. arborescens* and *A. barbadensis* on some opportunistic bacteria and fungi. Tested microorganisms are important human and animal pathogens and often represent groups of major infectious agents, i.e., Gram-positive bacteria (*B. cereus, S. aureus*), Gram-negative bacteria (*E. coli, P. aeruginosa,* and *P. fluorescens*), and yeasts (*C. albicans*). For all of these pathogens, a large increase in resistance to commonly used drugs was observed [43,44]. Many antimicrobial studies have been done in the last few years, mainly with *A. barbadensis*. Recently, Forno-Bell et al. [45] showed antibacterial activity of methanolic extract of *A. barbadensis* on *S. aureus, E. coli*, *Streptococcus uberis*, and MRSA cells. Next, Haque et al. [46] studied and proved antibacterial activity of *A. barbadensis* gel ethanol extract against *E. coli, Klebsiella pneumoniae, P. aeruginosa*, and *S. aureus*. Ben Moussa et al. [47] investigated antimicrobial effectiveness of different extract of *A. barbadensis* gel against foodborne pathogens. Ethanol extract did exhibit best results as its inhibited growth of *P. aeruginosa*, *S. aureus,* and *Aspergillus niger*.

The objective of our research was to investigate the content of bioactive phytoconstituents (total phenols, proanthocyanidins, proteins), enzymatic (amylase, cellulase, lipase, peroxidase, protease, transglutaminase), and antioxidant activities of fresh and lyophilized *A. arborescens* and *A. barbadensis* and their ethanol extracts. Furthermore, research of antimicrobial effectiveness of lyophilized *A. arborescens* and *A. barbadensis* and their ethanol extracts was performed, since gel and juice of aforementioned aloe species already showed good inhibitory efficacy [1]. Growth inhibition of both Gram-positive and Gram-negative bacteria was demonstrated using ethanolic extracts of *A. arborescens* and *A. barbadensis* as inhibitors by the disk diffusion method alone. A study on comparison of unknown *A. arborescens* and well-known *A. barbadensis* has been done.

This is the first study in which the activity of various enzymes has been determined, and it is important to emphasize the activity of cellulase and transglutaminase in all aloe samples. Moreover, it is the first study to quantify the antimicrobial activity of lyophilized aloes and their extracts. An important contribution to science is also the excellent inhibition of the growth of microorganisms with the addition of the less known lyophilized *A. arborescens* and its extract. The diversity of our samples and exceptional antimicrobial efficacy of these prove the versatility of using both aloes as antimicrobial agents.

## 2. Results

### 2.1. Efficacy of Lyophilization and Extraction of A. arborescens and A. barbadensis

As can be seen from Table 1, 99.4% of water was removed from both types of *Aloe* spp. The lyophilization process was successful, as sources indicate that *A. barbadensis* contain approx. 99.3% of water [26].

Furthermore, extracts with bioactive substances from *A. arborescens* and *A. barbadensis* were obtained by Soxhlet extraction. Table 1 shows the extraction efficiency. The yield was slightly higher in the extraction of *A. arborescens* (0.233 g of extract/g of DW) than *A. barbadensis* (0.142 g of extract/g of DW).

### 2.2. Phenolics and Proanthocyanidins Content, Total Protein Concentration, and Antioxidant Activity of A. arborescens and A. barbadensis

The contents of bioactive compounds present in *A. arborescens* and *A. barbadensis* were determined by spectrophotometric methods. The results are presented in Table 2.

The highest concentration of total phenols (TP) was detected in the ethanolic extract of *A. barbadensis*, while the extract of *A. arborescens* contained about five times lower TP content. The TP content in fresh and lyophilized samples was not detected as the concentrations were too low. Results are comparable to study from Vidic et al. [48], but they determined lower TP content in Soxhlet ethanol extract of *Aloe* spp. gel. The difference is probably due to the different preparation of the extracts, as we used lyophilized gel for extraction and determined higher TP content.

Like the TP content, the highest proanthocyanidin (PAC) content was detected in *A. barbadensis* extract. The PAC content is also about six times higher in *A. barbadensis* than in *A. arborescens*. Low concentrations of PAC were also detected in lyophilized samples, while they were not detected in fresh ones.

In terms of protein content, the highest concentration was present in the lyophilized samples, followed by ethanol extracts. Comparing the two aloes, *A. arborescens* showed highest total protein concentration in both cases.

Regarding antioxidant activity, fresh and lyophilized samples showed low percentage of inhibition, while ethanol extracts of *A. arborescens* and *A. barbadensis* showed a rapid decrease in absorbance and further highest percentage of inhibition were observed. The results for both aloes are similar and are completely comparable to already published studies [49]. Both ethanol extracts showed significant antioxidant activity.

The process of extracts preparation can also have a significant influence on the content of bioactive substances, as the degradation of thermally sensitive substances can also occur during Soxhlet extraction. Many factors, such as growing conditions, plant age, plant type, extraction processes, and analytical methods, can affect the presence of secondary metabolites in a plant and their biological activities [50]. Therefore, comparison of results from different studies is sometimes difficult or even impossible.

### 2.3. Enzymatic Activities of A. arborescens and A. barbadensis

Enzymes are highly specific biocatalysts involved in biotechnology. Plants are an important source of enzymes, especially those that are not naturally present in the human body (e.g., cellulase). Therefore, a study of various enzyme activities in different samples of *A. arborescens* and *A. barbadensis* was performed.

The activity of selected enzymes was determined in fresh and lyophilized gel of *A. arborescens* and *A. barbadensis* as well as in their ethanol extracts obtained by Soxhlet extraction. The stability of enzymes under extreme conditions (e.g., high temperature, pressure, solvent etc.) and the effect of medium factors are interesting for different industrial applications. Changes in protein structure may occur under extreme conditions. The spatial structure of many proteins may be significantly altered, causing denaturation and consequent loss in the activity. If conditions are less adverse protein structure may largely be retained. Minor structural changes may induce an alternative active protein state, which may possess altered activity, specificity, and stability [51,52]. The thermostability of enzymes is conditioned also by contributing factors, such as hydrogen bonds (intra- and intermolecular hydrogen bonds), electrostatic interactions, disulfide bonds, hydrophobic interactions, metal binding, deletion or shortening of loops, etc. However, there are no specific rules for thermostability of the enzymes. Among others, it may depend on the source from which the enzyme is derived [53]. The results of selected enzyme activities are presented in Table 3.

The highest α-amylase activity was present in the ethanol extract of *A. arborescens* and was almost four times higher than the activity in the ethanol extract of *A. barbadensis*. Comparing aloes, cellulase activity was higher in *A. arborescens* regardless of the type of the sample (fresh, lyophilized, extract), while lipase activity was only detected in lyophilized *A. barbadensis* and in its ethanol extract. Peroxidase activity was determined in all samples with higher value in *A. barbadensis* samples. The activity of the protease enzyme was determined in lyophilized *A. arborescens* and *A. barbadensis* and their extracts. The highest activity was achieved in the ethanol extract of *A. barbadensis*. The presence of the enzyme transglutaminase, which is particularly important in wound healing [54], was also detected in samples of both types of aloe. Slightly higher activity was present in *A. arborescens* samples. It should be emphasized that the difference in enzyme activities may appear due to the type of sample (fresh, lyophilized, extract), as the enzyme activity can be maintained or even increased by the lyophilization process [55].

The results of the study are remarkable, as plant materials such as aloe could be used to isolate enzymes for further applications. For example, α-amylase has gained much attention in recent years due to its ability to hydrolyze starch, which allows the inclusion of this enzyme in many applications, including in the baking industry, in environmentally friendly and safe detergents, and in the production of fructose syrup [56]. A particularly important contribution to the study is also the generally high cellulase activity in all samples, especially in the ethanolic extract of *A. arborescens*, as cellulase is an extremely applicative enzyme. The key areas of cellulase application in industry are currently beverages, detergents, food, healthcare, paper, and textiles. The potential of cellulases in the fight against antibiotic-resistant bacteria is also extremely interesting [57]. On the other hand, lipases isolated from plants represent potential for commercial applications in the food, detergent, and pharmaceutical industries, but their low expression in plants and difficulty in isolation limit their commercial applicability [58]. Furthermore, peroxidases are one of the key antioxidant enzymes used in the fields of environment, medicine, agriculture, and analytics. One of the more widespread applications is the use of horseradish peroxidase in the development of biosensors [59], while plant proteases are mostly used in bioactive peptide production, baking industry, dairy processing, and meat tenderization [60]. In addition to the previously mentioned important role of transglutaminase in wound healing, some research has also emerged on the potential use of plant transglutaminase as a food additive [61]. To the best of our knowledge, no similar comparative study in the literature that contains enzymatic activities in different samples of *A. arborescens* and *A. barbadensis* was found. Therefore, the obtained results present a major contribution to the identification of important enzymes from *Aloe* spp.

### 2.4. Qualitatively Determined Antimicrobial Activity of A. arborescens and A. barbadensis

Using the disk diffusion method on nutrient agars, the inhibitory property of lyophilized aloes and their ethanol extracts was qualitatively determined. Tested pathogenic microorganisms cause various infections, e.g., *Candida* fungus is one of the most common causes of fungal infections (candidiasis) and in addition, it is one of the most often tested on cosmetic products, besides *E. coli*, *S. aureus,* and *P. aeruginosa* [62,63].

The disk diffusion method showed the antimicrobial efficacy of *A. arborescens* and *A. barbadensis* only in the case of ethanol extracts. Lyophilized samples did not indicate inhibitory properties as no inhibition zone was detected.

Ethanol extracts of both aloes showed inhibitory properties at Gram-positive bacteria *B. cereus* and Gram-negative bacteria *E. coli* and *P. fluorescens*, while the growth of other tested microbial (*C. albicans, P. aeruginosa, S. aureus*) cultures was not inhibited. Table 4 shows the microbial growth inhibition zone in the case of using ethanol extracts of *A. arborescens* and *A. barbadensis* as inhibitors.

From Table 4, it can be seen that the growth of *E. coli* was better inhibited by the ethanol extract of *A. barbadensis* than in the case of *A. arborescens* at both initial concentrations (10^6^ and 10^7^ CFU/mL). Comparing our results to other studies, their ethanol extract of *A. barbadensis* did not show any inhibition [16,20], or the growth inhibition was much lower [34]. Growth inhibition of *P. fluorescens* was perceived in other studies only in the case of *A. barbadensis* methanol extract which contained aloe emodin and has already been shown to have antimicrobial effect [3]. In our study, both ethanol extracts were confirmed to be good antimicrobial agents. The growth of *P. fluorescens* was slightly better inhibited by the ethanol extract of *A. arborescens* with somewhat larger inhibition zone. At higher initial concentration (10^7^ CFU/mL) of *P. fluorescens*, the inhibition zone was not observed. In the case of *B. cereus* growth, the inhibition zone was equal for both aloes regardless of the initial concentration, meaning that they are equally effective antimicrobial agents for this microorganism. The results are comparable to the study, where an inhibition zone larger than 8 mm at higher initial concentration of microorganism was determined [34]. 

### 2.5. Quantitatively Determined Antimicrobial Activity of A. aborescens and A. barbadensis

**Figure 2:**
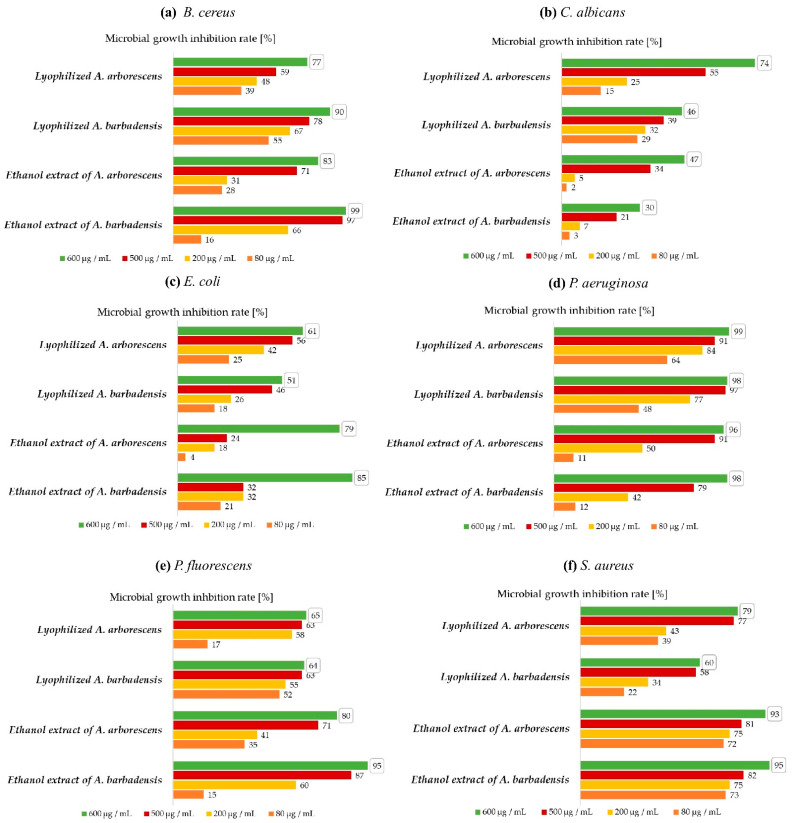
MGIRs for lyophilized aloe and their ethanol extracts using 600, 500, 200, and 80 μg of sample/mL of suspension; (**a**) MGIRs for *B. cereus*; (**b**) MGIRs for *C. albicans*; (**c**) MGIRs for *E. coli*; (**d**) MGIRs for *P. aeruginosa*; (**e**) MGIRs for *P. fluorescens*; (**f**) MGIRs for *S. aureus*; Initial concentrations of microbial cultures: *B. cereus* 10^7^ CFU/mL, *C. albicans* 10^6^ CFU/mL, *E. coli* 10^7^ CFU/mL, *P. aeruginosa* 10^7^ CFU/mL, *P. fluorescens* 10^7^ CFU/mL, and *S. aureus* 10^5^ CFU/mL. The numbers in boxes indicate the highest MGIR for specific sample. Data expressed as mean ± standard deviation of three replicates that vary for max. 3%.

Since there is a lack in studies that would contain quantitative antimicrobial activity, *A. arborescens* and *A. barbadensis* were further tested by the broth microdilution method. This offers quantitative results to determine the microbial growth inhibition rate (MGIR) at different concentrations of the added antimicrobial sample. In our study, samples used as antimicrobial agents were ethanol extracts from *A. arborescens* and *A. barbadensis*, as well as lyophilized aloes. The inhibitory efficacies of *A. arborescens* and *A. barbadensis* on the growth of microbial cells were tested at a specific initial concentration (see Figure 2 caption for details) of each microorganism.

#### 2.5.1. Lyophilized *A. arborescens* and *A. barbadensis*

By lyophilization, the moisture content of *A. arborescens* and *A. barbadensis* was reduced to a minimum (99.4% of water was removed) while no thermosensitive substances were destroyed, and most importantly, biological activity of the compounds present in lyophilized samples was mantained. Due to the lyophilization process, the sample is more stable because no water is present. These advantages are also exploited in the storage of samples. Very few studies [16,35,64] on the antimicrobial activity of lyophilized *A. arborescens* and *A. barbadensis* were described in the reviewed literature, and thus, the study of the antimicrobial efficacy of lyophilized aloes was performed in our research. Figure 2 shows MGIR for *B. cereus, C. albicans, E. coli, P. aeruginosa, P. fluorescens,* and *S. aureus* in the case of using lyophilized *A. arborescens* and *A. barbadensis* as inhibitors.

Both lyophilized *A. arborescens* and lyophilized *A. barbadensis* (600 μg sample/mL suspension) inhibited the growth of *P. aeruginosa* most effectively. The lowest MGIR for lyophilized *A. arborescens* sample was shown in the case of *E. coli* (61 ± 1% MGIR) and the lyophilized *A. barbadensis* sample by *C. albicans* (46 ± 1% MGIR). The growth of *B. cereus* was better inhibited by lyophilized *A. barbadensis* (90 ± 2% MGIR) than by *A. arborescens* (77 ± 1% MGIR). Quite a difference was shown in the inhibition of the growth of the yeast *C. albicans*, as the lyophilized *A. arborescens* inhibited its growth with 74 ± 2% MGIR while the lyophilized *A. barbadensis* with only 46 ± 1% MGIR. Regarding growth inhibition of *E. coli*, lyophilized *A. barbadensis* exhibited 51 ± 3% MGIR, which is lower than beforementioned MGIR in the case of *A. arborescens*. The growth inhibition of *P. aeruginosa* is particularly prominent, as the samples almost completely (99 ± 1% MGIR at *A. arborescens* and 98 ± 1% MGIR at *A. barbadensis*) inhibited the growth of their strains. The growth of *P. fluorescens* was equally inhibited by lyophilized *A. arborescens* and *A. barbadensis*. In a recent study, Habeeb and others [33] determined a minimum inhibitory concentration (MIC) of lyophilized *A. barbadensis* 25000 μg/mL at the initial concentration 10^5^ CFU/mL of the *S. aureus* microorganism. In our study, at the same initial concentration of *S. aureus*, a 60 ± 1% MGIR was achieved, with the addition of 600 μg/mL lyophilized *A. barbadensis*, which proves that a lower concentration of lyophilized *A. barbadensis* can already inhibit the growth of *S. aureus*. *A. arborescens* inhibited the growth of *S. aureus* strains even more effectively (79 ± 2% MGIR).

Regarding the inhibition of microbial cells at lower concentrations of added inhibitory agent, the effect of *A. arborescens* and *A. barbadensis* on the growth of *P. aeruginosa* should be emphasized. The addition of 500 μg/mL of lyophilized aloes achieved above 90% MGIR. Moreover, the growth of *P. aeruginosa* was impaired with the addition of even lower concentrations.

Both lyophilized aloes seem to be good antimicrobial agents. In addition to the determination of MIC values for *S aureus* [33], other MIC determinations for lyophilized aloes were not found in the reviewed literature. Based on the obtained results, the MIC_90_ value for lyophilized *A. barbadensis* in the case of *B. cereus* was 600 μg/mL, while both lyophilized samples showed exceptional antibacterial efficacy on *P. aeruginosa*. The MIC_90_ value for *A. arborescens* was 457 μg/mL and for *A. barbadensis* 395 μg/mL. For other microbial cells, further studies with added higher sample concentrations would be required to determine the MIC values. In general, *A. arborescens* showed in common better microbial growth inhibition because of a higher MGIR for all microbial cultures except *B. cereus*.

Quantification of the MGIR at four different concentrations of lyophilized samples of *A. arborescens* and *A. barbadensis* against fungi, Gram-positive and Gram-negative bacteria, has not been performed in any study to date.

#### 2.5.2. Ethanol Extracts of *A. arborescens* and *A. barbadensis*

Most published research on the topic of antimicrobial activity of aloes involves testing different extracts as inhibitors. In our study, we used ethanol as a solvent in the extraction process as it is known to be effective, efficient, and safe.

Figure 2 shows MGIR in the case of using ethanol extracts of *A. arborescens* and *A. barbadensis* as inhibitors. Ethanol extracts of *A. arborescens* and *A. barbadensis* (600 μg of sample/mL of suspension) showed an extremely high MGIR in the case of all tested microbial cells. The highest inhibition of *A. arborescens* ethanol extract was detected for the growth of *P. aeruginosa* (96 ± 3% MGIR) and the lowest for the growth of *C. albicans* (47 ± 2% MGIR), respectively. Ethanol extract of *A. arborescens* also inhibited growth of *E. coli* with 79 ± 2%, *P. fluorescens* with 80 ± 3%, *B. cereus* with 83 ± 2%, and *S. aureus* with 93 ± 1% MGIR. Ethanol extract of *A. barbadensis* gave the highest inhibition for growth of *B. cereus* (99 ± 1% MGIR) following for the growth of *P. aeruginosa* (98 ± 2% MGIR). Moreover, *A. barbadensis* extract inhibited *S. aureus* and *P. fluorescens* with 95 ± 1% MGIR. Growth of *E. coli* was inhibited with 85 ± 2% MGIR and growth of *C. albicans* with 30 ± 3% MGIR.

In the case of ethanol extract as an inhibitor, *A. barbadensis* extract was generally slightly better than *A. arborescens* as it achieved higher inhibition rate for almost all tested microorganisms (except *C. albicans*, where the MGIR was only 30 ± 3%).

Therefore, it is interesting to compare the results at a lower added concentration of *A. arborescens* and *A. barbadensis* ethanol extracts. Since MGIRs with the addition of 500 μg/mL ethanol extract were still high especially for *B. cereus, P. aeruginosa, P. fluorescens,* and *S. aureus*, it is important to consider the results of the MGIR with the addition of 80 and 200 μg sample/mL suspension.

The results of our antimicrobial efficacy study of *A. arborescens* and *A. barbadensis* ethanol extracts show that both samples at least slightly inhibited the growth of all microbial cells, with the addition of 200 μg/mL inhibitory agent. Ethanol extract of *A. arborescens* with the concentration of 80 μg/mL did not inhibit the growth of *E. coli* and *C. albicans*, and ethanol extract of *A. barbadensis* did not inhibit the growth of *C. albicans* strains. In both cases, ethanol extracts proved to be the strongest antimicrobial agents among all tested samples in inhibiting the growth of *S. aureus*, as they achieved 72 ± 3% and 73 ± 1% MGIR at a concentration of 80 μg/mL, respectively.

Already published studies provide different MIC values when using ethanol extracts of *A. barbadensis* as antimicrobial agents. MIC values for *E. coli* are in the range of 10000-125 μg/mL [22,38,46], for *S. aureus* between 500-125 μg/mL [22,38,46], for *P. aeruginosa* between 650-100 μg/mL [22,41,46], and for *C. albicans* strains about 400 μg/mL [38]. MIC values may differ between studies mainly due to different ethanol extract preparation procedures. In most cases, however, the initial concentrations of microbial cultures are unknown. The MIC values for *B. cereus* and *P. fluorescens* were not found in the literature reviewed, while MIC_90_ values for the mentioned microorganisms were successfully determined in our study.

The MIC_90_ value for the ethanol extract of *A. barbadensis* in the case of *B. cereus* is 432 μg/mL and in the case of *P. fluorescens* is 538 μg/mL. Higher concentrations should be tested to determine the MIC value for ethanol extract of *A. arborescens*. A MIC_90_ value was also determined for both extracts in the case of *P. aeruginosa* (493 μg/mL for *A. arborescens* and 558 μg/mL for *A. barbadensis*) and *S. aureus* (575 μg/mL for *A. arborescens* and 562 μg/mL for *A. barbadensis*).

While some antimicrobial studies of *A. barbadensis* extracts can be found in the literature, the quantification of antimicrobial efficacy of *A. arborescens* extract is a major contribution to this research field. In general, the ethanol extract of *A. barbadensis* had a slightly more effective growth inhibition against all microorganisms except on yeast *C. albicans*, where the higher MGIR has been reached with the ethanol extract of *A. arborescens*.

## 3. Discussion

A comparative study of different samples of *A. arborescens* and *A. barbadensis* was performed.

Numbers of various secondary metabolites are found in plants which contribute to compelling biological activities. Our study shows the presence of different important phytoconstituents in *A. arborescens* and *A. barbadensis*. Ethanol extract for which the contents of total phenols and proanthocyanidins were determined also showed good antioxidant activity. In various studies [65,66,67,68], a number of potential antioxidant and antimicrobial components have been isolated from *Aloe* species, which most likely contribute to the biological activity of *Aloe* with a synergistic effect. Additional studies with LC-MS have also been previously performed, where mostly anthraquinones, phytosterols, alkaloids, and fatty acids were identified [69,70].

The presence of enzymes in *A. arborescens* and *A. barbadensis* was also demonstrated. Extracts and lyophilized samples in particular showed higher enzyme activities. The presence of the enzyme transglutaminase, which is involved in stabilization, general physiology, and repair of many areas of tissue (e.g., skin) [54], should be emphasized. The presence of transglutaminase in all samples proves that aloe can contribute to better and faster wound healing [71].

Using a qualitative disk diffusion method, the antibacterial efficacy on the growth of both Gram-positive and Gram-negative bacteria of ethanol extracts was determined. Further, antimicrobial activity of *A. arborescens* and *A. barbadensis* was determined quantitatively. Interestingly all of *A. arborescens* samples showed the highest inhibition for the growth of *P. aeruginosa*, as did the lyophilized *A. barbadensis* sample. Furthermore, ethanol extract of *A. barbadensis* gave the highest inhibition for the growth of *B. cereus*.

Previously, various preparations of *A. vera* including creams, juices, and gels have been used as a traditional medicine in some parts of the world to treat various diseases [72]. The fact that *A. arborescens* and *A. barbadensis* are effective antimicrobial agents was also reinforced by performed study. *A. arborescens* and *A. barbadensis* are possible solution as antimicrobials for different applications in food production. For example, their extracts or potentially isolated antimicrobial compounds can be incorporated in different package materials to prevent or inhibit microbial growth and, moreover, can be extensively used in cosmetic and pharmaceutical products, as in natural food and dietary supplements. Additionally, the combination of antimicrobial efficacy and the presence of transglutaminase in the obtained samples show the even greater potential of aloe preparations in medical application.

*E. coli,* found in the human gastrointestinal tract, lives in a mutually beneficial relationship with the host, but it is also one of the most common pathogens in humans as it is responsible for a wide range of diseases [73,74]. It is a food-borne pathogen, and with the extensive use of antibiotics, food-borne pathogens develop antibiotic resistance which means many food-borne illnesses and thus a lack of effective treatment [75]. The best inhibitory properties for *E. coli* growth have been found to have *A. barbadensis* ethanol extract and right after there is *A. arborescens* ethanol extract. Comparing results with our published study (with fresh gels and juices from aloes) [1], better antimicrobial efficacy on *E. coli* growth displayed all *A. barbadensis* samples (except lyophilized).

Although fresh juice of *A. barbadensis* and *A. arborescens* does not inhibit the growth of *S. aureus* [1], an excellent inhibition for the same microbial species was found for ethanol extracts of both aloes. However, a better growth inhibitor of most selected microorganisms was the extract of *A. barbadensis*; lyophilized *A. arborescens*, however, offered better inhibition of *S. aureus* growth than lyophilized *A. barbadensis*. Multidrug resistance is a well-known problem in medicine, and *S. aureus* is perhaps the bacterium of the highest concern due to its virulence and ability to cause a diverse set of life-threatening infections [76,77]. Our research has shown that *A. arborescens* and *A. barbadensis* have a great potential in terms of reducing possible infections with *S. aureus* strains.

In addition, research showed all samples to be good growth inhibitors of *B. cereus,* a pathogenic spore-forming bacterium that is often associated with food-borne diseases as spores can also survive pasteurization and cooking and multiply when foods are stored incorrectly [78,79]. Best inhibitory properties for the growth of *B. cereus* showed *A. barbadensis* ethanol extract. Comparing the results of both aloes for inhibition of this microorganism, MGIRs of *A. barbadensis* were higher for all samples.

The tested samples from this study and our previous one [1] were found to be good inhibitors of *P. fluorescens* growth, which is opportunistically pathogenic and able to reside in many environments [80]. The best inhibitory growth properties for this microorganism showed *A. barbadensis* ethanol extract. A comparison of the inhibitory effect of both aloes shows that *A. barbadensis* samples have a better antimicrobial effect on *P. fluorescens* growth than *A. arborescens* samples. Lyophilized samples and fresh gels of both aloes have a similar inhibitory effect on growth of *P. fluorescens*. In common, all tested inhibitors showed satisfactory results for the *P. fluorescens* growth inhibition study.

Further, the best inhibitory properties for the growth of the yeast *C. albicans* have fresh *A. barbadensis* gel [1] and lyophilized *A. arborescens*. Comparing the results of both aloes, *A. barbadensis* showed to have a better antimicrobial effect on the growth of *C. albicans*, which is the fourth leading cause of bloodstream infections [81].

According to obtained results, all samples were also good inhibitors of *P. aeruginosa* growth. Therefore, *A. arborescens* and *A. barbadensis* could reduce the possible infections with *P. aeruginosa,* which has a leading role among infections caused by Gram-negative strains [82]. Due to its nutritional versatility, high number of virulence factors, and high antibiotic resistance, treatment is extremely difficult [83]. Both, *A. barbadensis* and *A. arborescens* gave high inhibition level for the growth of *P. aeruginosa* irrespective of the form of the sample (fresh, lyophilized, or ethanol extract) [1]. These results are very important as *P. aeruginosa* is one of the first three causes of opportunistic infections in humans [84].

For the first time, the enzymatic and antimicrobial activity of lyophilized *A. arborescens* and *A. barbadensis* and their extracts were quantitatively determined. A comprehensive study confirmed the presence of versatile enzymes in *A. arborescens* and *A. barbadensis* as well as the growth inhibition rates for six microorganisms, representatives of fungi, Gram-negative and Gram-positive bacteria, with the addition of four different concentrations of *A. arborescens* and *A. barbadensis,* as inhibitors. The results confirm and give credence to the beneficial effects of using *A. arborescens* and *A. barbadensis* and their extracts.

## 4. Materials and Methods

### 4.1. Chemicals and Reagents

Acetonitrile, agar, bovine serum albumin (BSA), casein, 3,5-dinitrosalicylic acid (DNS), 2,2-diphenyl-1-picrylhydrazyl (DPPH), Folin-Ciocalteu’s Phenol-reagent (FC), gallic acid (GA), glucose assay, hydrochloric acid, hydrogen peroxide, hydroxylamine hydrochloride (99%), L-glutamic acid γ-monohydroxamate, L- Glutathione reduced, maltose, methanol, yeast extract, peptone from soybean, phenol, p-nitrophenyl butyrate (p-NPB), potassium sodium tartrate tetrahydrate, Sigmacell cellulose, sodium acetate, sodium carbonate, starch, trichloroacetic acid (TCA), and Tris buffer (Trizma Base) were purchased from Sigma-Aldrich (St. Louis, USA). Chemicals including acetic acid (100%), 4-aminoantipyrine (4-APP), Coomassie Blue G-250, ethanol, ferric chloride, hydrogen chloride (HCl), meat extract, meat peptone, n-butanol, phosphoric acid (85%), potassium dihydrogen phosphate, sodium chloride, sodium dihydrogen phosphate monohydrate, and sodium hydrogen phosphate were obtained from Merck (Darmstadt, Germany). Calcium chloride, D-(+)-glucose anhydrous, and iron(II) sulfate heptahydrate were purchased at Kemika (Zagreb, Croatia), Mueller–Hinton broth and potato dextrose agar were from Biolife (Milano, Italy). Malt extract, potato dextrose broth, Triton X-100, tryptic soy broth, and tryptone were purchased from Fluka (Buchs, Switzerland). CBZ-Glutaminylglycine (Z-Gln-Gly) was purchased from Zedira GmbH (Darmstadt, Germany).

### 4.2. Plant Material and Preparation of Samples

The gels of *A. arborescens* and *A. barbadensis* were obtained from fresh mature leaves of *A. arborescens* and *A. barbadensis*. For one batch, 5 leaves (345.2 g of *A. arborescens* and 390.7 g of *A. barbadensis*) of each aloe, long between 50-60 cm, were washed under running water and thick outer layers of the leaves were separated using a knife. 165.3 g of *A. arborescens* and 214.1 g of *A. barbadensis* inner cores were cut into approx. 1 cm^3^ big pieces and centrifuged (Eppendorf^®^ Centrifuge 5840R, Wesseling, Deutschland) at 11,000 rpm, room temperature for 15 minutes. The supernatant (juice) was removed and 149.8 g of *A. arborescens* and 197.5 g of *A. barbadensis* fresh transparent gel was collected and homogenized (Tehtnica^®^ Rotamix 701 MD, Železniki, Slovenia). For one batch, 148.5 g of fresh *A. arborescens* gel and 196.6 g of fresh *A. barbadensis* gel were subjected to a lyophilization process Kambič^®^ Freeze Dryer LIO 2000 PNS, Semič, Slovenia) to remove water. Thus, lyophilized *A. arborescens* (0.9 g DW) and *A. barbadensis* (1.2 g DW) were obtained. As the samples of lyophilized aloes were too dry to apply, they were diluted with a minimal amount of distilled water before use and homogenized. Lyophilized *A. arborescens* and *A. barbadensis* were always prepared at a concentration of 0.1 g/mL.

Furthermore, using the Soxhlet apparatus, extractions of lyophilized gel of *A. arborescens* and *A. barbadensis* were performed. For one batch, the lyophilized gel of *A. arborescens* (0.9 g DW) or *A. barbadensis* (1.2 g DW) was placed in a porous bag made from a strong filter paper, which was placed in Soxhlet extractor. A volume of 150 mL of ethanol was used as an extraction solvent. The solvent was further evaporated using rotavapor (Büchi^®^ Rotavapor R-144, Flawil, Switzerland) and ethanol extracts of *A. arborescens* (0.21 g) and *A. barbadensis* (0.17 g) were obtained. Extracts were stored at 4 °C until use. Ethanol extracts of *A. arborescens* and *A. barbadensis* were always prepared at a concentration of 0.1 g/mL in 5% DMSO.

### 4.3. Determination of Total Phenolics (TP) Content

The content of TP was determined using Folin–Ciocalteu’s reagent. A volume of 0.5 mL of the prepared sample solution (2 g/L) was mixed with 2.5 mL of FC solution, previously diluted with distilled water in 1:10 ratio. Further, 2 mL of Na_2_CO_3_ solution with concentration of 75 g/L was added to each sample; samples were then incubated for 5 min in a water bath at 50 °C. The solutions were cooled to room temperature, and the absorbance was measured at 760 nm. Following a similar procedure, a standard curve with gallic acid was prepared. The results are expressed as mg of GA per g of sample. The experiments were performed in triplicates, and the results represent the mean values and standard deviations.

### 4.4. Determination of Proanthocyanidins (PAC) Content

The PAC content was determined by the calorimetric method using hydrochloric acid and n-butanol. To 1 mL of the prepared sample solutions (5 mg/mL), 10 mL of FeSO_4_ × 7 H_2_O in a mixture of HCl and n-butanol (2:3) was added. The prepared solutions were incubated for 15 min in a water bath at 95 °C. The absorbance of the cooled samples was measured at 540 nm. Based on the measured absorbance, the mass concentration of PAC was calculated, expressed as mg of PAC per g of sample. All experiments were performed in triplicates, and the results represent the mean values and standard deviations.

### 4.5. Determination of Antioxidant Activity

Antioxidant activity was determined using DPPH method [65]. A 77 μL of prepared sample solutions (1 mg/mL) and 3 mL of a DPPH solution prepared in methanol (6 ×10^−5^ M) were mixed. The solutions were then incubated for 15 min at room temperature in the dark, and the absorbance at 515 nm was immediately measured. Antioxidant activity is expressed as a percentage of inhibition relative to the reference solution, containing 77 μL of methanol and 3 mL of prepared DPPH solution [85]. All experiments were performed in triplicates, and the results represent the mean values and standard deviations.

### 4.6. Determination of Total Protein Concentration

The total protein concentration in *A. arborescens* and *A. barbadensis* samples was determined by Bradford method [86] using bovine serum albumin as a standard. A volume of 1 mL of Bradford reagent was pipetted into a microcentrifuge, and 20 μL of sample was added. The solutions were stirred immediately and incubated for 15 min at room temperature; then, their absorbance at 595 nm was measured. Following a similar procedure, a standard curve with BSA was prepared. The results are expressed as mg of proteins per g of sample. All experiments were performed in triplicates, and the results represent the mean values and standard deviations.

### 4.7. Determination of Enzyme Activities

Activities of selected enzymes were defined using specific spectrometric activity assays. All experiments were performed in triplicates.

- α-Amylase activity was determined by the DNS method [87] with starch as the substrate and maltose as the standard. A volume of 0.5 mL of prepared sample solutions and 0.5 mL of 1% (*w/v*) starch solution prepared in sodium buffer solution was pipetted into suitable centrifuge tubes. Mixture was incubated for 3 min at 20 °C; then, color reagent (prepared with 5.3 M potassium sodium tartrate, tetrahydrate, and 96 mM DNS solution) was added. Covered containers were incubated in a boiling water for 15 min. A volume of 10 mL of distilled water was added to cooled solutions and mixed by inversion. The absorbance was measured at 540 nm. Results are expressed as units per gram of sample; one unit will liberate 1 mg of maltose from starch in 3 min at 20 °C at pH 6.9.
- Cellulase activity was measured using glucose as substrate [88]. A volume of 4 mL of a Sigmacell solution was pipetted into suitable containers, then 1 mL of sample was added. Mixture was incubated for 120 min at 37 °C with moderate shaking. Further, suspension was transferred into iced water bath. When suspension was settled, it was centrifuged at 11,000 rpm for 2 min, and 100 μL of supernatant was added to 3 mL of glucose solution. Absorbance was measured at 340 nm for 5 min, and the increase in absorbance was used to determine enzyme activity. Results are expressed as units per gram of sample; one unit liberates 1 μmol of glucose from cellulose in one hour at 37 °C and pH 5.0.
- Lipase activity was determined using p-NPB as substrate [89]. A volume of 0.9 mL of 100 mM sodium phosphate buffer with 150 mM sodium chloride and 0.5% triton was pipetted into suitable containers. A volume of 0.1 mL of sample was added, and the mixture was incubated for 5 min at 37 °C. Further, 0.01 mL of 50 mM p-NPB was added, and absorbance was measured at 400 nm for 5 min. The increase in absorbance was used to determine enzyme activity. The results are expressed as units per gram of sample; one unit will release 1 nmol of p-nitrophenol per minute at 37 °C and pH 7.2 using p-NPB.
- Peroxidase activity was determined using H_2_O_2_ as an inhibitor [90]. A volume of 1.4 mL of solution of 0.0025 M 4-APP with 0.17 M phenol was added into suitable containers, and 1.5 mL of 0.0017 M H_2_O_2_ and 0.1 mL of sample was added. Solution was mixed, and absorbance was immediately measured at 510 nm for 4 min. The results are expressed as units per gram of sample; one unit will decompose one μM of H_2_O_2_ per minute at 25 °C at pH 7.0.
- Protease activity was determined using casein as substrate [88]. A volume of 1 mL of casein solution prepared in phosphate buffer was incubated for 3 min at 35 °C. Then 0.5 mL of phosphate buffer and 0.5 mL of sample was added. Mixture was incubated for 20 min at 35 °C. After incubation, 3 mL of 5% (*v/v*) TCA was added and further incubated for 30 min at room temperature. Mixture was centrifuged at 6000 rpm for 20 min, and the absorbance of the obtained supernatant was measured at 280 nm. Results are expressed as Tu^cas^ g^−1^, which represents amount of casein hydrolyzed per g of sample per minute [91].
- Transglutaminase activity was determined with a colorimetric method [92] using hydroxylamine as amine donor and Z-Gln-Gly as substrate. A volume of 20 mL of reaction cocktail was mixed with 30 μL of sample solution at 37 °C for 10 min. Then, 0.5 mL of 12% (*v/v*) TCA solution was pipetted, mixed, and finally, 0.5 mL of 5% (*w/v*) ferric chloride solution was added. Mixture was centrifuged for 5 min. Absorbance of supernatants was recorded at 525 nm. Results are expressed as units per gram of sample; one unit form 1 μmole of hydroxamate per minute at 37 °C and pH 6.0.

### 4.8. Determination of Antimicrobial Activity

#### 4.8.1. Microorganisms

The antimicrobial activity of various inhibitory samples of *A. arborescens* and *A. barbadensis* was detected against several pathogenic microbes, including bacteria (*Escherichia coli* DSM 498, *Staphylococcus aureus* DSM 346, *Bacillus cereus* DSM 345, *Pseudomonas fluorescens* DSM 289, *Pseudomonas aeruginosa* DSM 1128) and fungi (*Candida albicans* DSM 1386). Standard strains were purchased from DSMZ-German Collection of Microorganisms and Cell Cultures GmbH (Braunschweig, Germany).

#### 4.8.2. Disc Diffusion Method

The disk diffusion method [93] was used to qualitatively determine the susceptibility of microorganisms to different antimicrobial samples. The antimicrobial efficacy of *A. arborescens* and *A. barbadensis* on bacteria and fungi were performed at optimal conditions and two initial concentrations of each microorganism as in our previous study [1]. The indicator of the inhibitory property of the sample was the inhibition zone shown at the sample. The diameter of the growth inhibition zone was measured to compare the antimicrobial efficacy of the two aloes on the growth of different microbial cultures. dH_2_O and 5% DMSO were used as negative controls. Vancomycin and amoxicillin (30 μg/disc) were used as positive controls. All experiments were performed in triplicates.

#### 4.8.3. Broth Microdilution Method

A broth microdilution method [94] was used to quantitatively analyze and thereby determine the microbial growth inhibition rate (MGIR) at different sample concentrations (80, 200, 500, and 600 μg of sample/mL of microbial suspension). MGIRs were determined based on optical density of the growth control and sample to determine the percentage of microbial growth inhibition [1]. Additionally, MIC_90_ values were determined experimentally or calculated, as concentrations where the samples inhibited the growth of microbes by 90% MGIR [1]. All experiments were performed in triplicates.

## 5. Conclusions

The results of our research demonstrated the presence of bioactive substances such as phenolics, proanthocyanidins, and enzymes in samples of *A. arborescens* and *A. barbadensis*. Particularly more concentrated samples, such as extracts or lyophilized ones, are rich in biologically active ingredients and exhibit high antioxidant potential. Furthermore, the results have proved *A. arborescens* and *A. barbadensis* to hold excellent potential as antimicrobial agents. Both aloes inhibited the growth of *B. cereus, C. albicans, E. coli, P. aeruginosa, P. fluorescens*, and *S. aureus*, representatives of Gram-positive, Gram-negative bacteria, and fungi. Further, for most tested microorganisms (*B. cereus, E. coli, P. fluorescens, S. aureus*) the best inhibitory effect was found for ethanol extracts of *A. barbadensis* and *A. arborescens*. The best inhibitory properties of the tested samples (even the fresh ones) were shown for *P. aeruginosa* growth. *P. aeruginosa* can cause a wide range of infections (respiratory tract, urinary tract, skin infections, superficial structures of the eye, etc.), prevailing infections of wounds and burns. According to the obtained results, the use of personal hygiene products such as soaps and various creams containing *A. arborescens* and *A. barbadensis* could reduce the possible infections with *P. aeruginosa* and other tested microorganisms.

As both aloes are rich in essential and bioactive nutrients as phytochemicals, enzymes, and other compounds, they need to be utilized as much as possible. Functional foods and food supplements from natural sources like *A. arborescens* and *A. barbadensis*, are a good possibility to intake essential and bioactive molecules and nutrients in the human body since they are natural and not synthesized and therefore more receptive to the human body. Further, incorporation of both *A. arborescens* and *A. barbadensis* into different materials as antimicrobials could be used for applications in drug delivery, wound healing, etc. Such materials have a high potential to reduce microbial growth, and they could make a huge contribution to the food, medicinal, and pharmaceutical industries. Furthermore, characterization and isolation of individual bioactive constituents from *A. vera* extracts should be performed to determine the antimicrobial efficacy of the individual components. In addition, toxicity studies of *A. vera* extracts should be performed to determine the safety indices of the extracts. Clinical trials should also be conducted to investigate the potential of *A. vera* extracts in the treatment of, e.g., bacterial diseases.

## Figures and Tables

**Table 1:** Mass balance of the lyophilization and extraction process.

Lyophilization	*A. arborescens*	*A. barbadensis*
Mass of fresh material used for lyophilization [g]	148.5	196.6
Mass of dried material obtained after lyophilization [g]	0.9	1.2
Moisture removed [%]	99.4	99.4
Dry matter [%]	0.6	0.6
**Extraction**		
Mass of dried material used for extraction [g]	0.9	1.2
Mass of obtained extract [g]	0.21	0.17
Extraction yield [%]	23.3	14.2
**Yield after lyophilization and extraction [%]**	0.14	0.09

**Table 2:** Bioactive compounds content in *A. arborescens* and *A. barbadensis* samples and their antioxidant activity.

Sample	Total Phenolic Content	Proanthocyanidin Content	Total Protein Concentration	Antioxidant Activity
	[mg/g] ^1,2^	[mg/g] ^3^	[mg/g] ^4^	[% inhibition] ^5^
*A. arborescens* gel	-	-	0.94 ± 0.24	5.54 ± 0.84
*A. barbadensis* gel	-	-	1.32 ± 0.09	5.46 ± 0.62
Lyophilized *A. arborescens*	-	0.01 ± 0.00	9.77 ± 1.22	8.31 ± 1.13
Lyophilized *A. barbadensis*	-	0.01 ± 0.00	6.06 ± 0.98	7.86 ± 1.58
Ethanol extract of *A. arborescens*	1.42 ± 0.15	0.22 ± 0.08	5.93 ± 0.43	61.55 ± 7.31
Ethanol extract of *A. barbadensis*	7.25 ± 1.04	1.35 ± 0.18	4.11 ± 1.07	59.29 ± 5.29

Note: ^1,3,4^ Data expressed per gram of sample. ^2^ Concentration based upon gallic acid as standard. ^5^ % DPPH radical scavenging activity. - not detected. All displayed results represent the mean value and standard deviation.

**Table 3:** Enzyme activities in *A. arborescens* and *A. barbadensis* samples.

Sample	α-amylase	Cellulase	Lipase	Peroxidase	Protease	Transglutaminase
	[U/g] ^1^					
*A. arborescens* gel	0.01 ± 0.00	413.75 ± 11.18	-	0.02 ± 0.01	-	0.39 ± 0.10
*A. barbadensis* gel	0.01 ± 0.00	56.49 ± 6.42	-	0.09 ± 0.01	-	0.15 ± 0.04
Lyophilized *A. arborescens*	0.21 ± 0.01	314.88 ± 14.95	-	0.21 ± 0.03	0.20 ± 0.09	1.46 ± 0.22
Lyophilized *A. barbadensis*	0.09 ± 0.01	245.36 ± 8.61	1.62 ± 0.04	0.94 ± 0.21	0.03 ± 0.01	0.86 ± 0.03
Ethanol extract of *A. arborescens*	21.51 ± 2.16	1165.34 ± 57.22	-	1.41 ± 0.19	1.15 ± 0.36	1.81 ± 0.24
Ethanol extract of *A. barbadensis*	5.59 ± 1.03	768.82 ± 29.16	36.03 ± 3.41	2.00 ± 0.54	2.31 ± 0.48	1.12 ± 0.18

Note: ^1^ Data expressed as units per gram of sample. - not detected. All displayed results represent the mean value and standard deviation.

**Table 4:** Microbial growth inhibition zone diameter using ethanol extracts of *A. arborescens* and *A. barbadensis* as inhibitors.

Microorganism	Concentration [CFU/mL]	Microbial Growth Inhibition Zone Diameter [mm]	
		Ethanol Extract of *A. Arborescens*	Ethanol Extract of *A. Barbadensis*
***E. coli***	10^6^	11 ± 0	13 ± 1
	10^7^	11 ± 0	12 ± 1
***B. cereus***	10^6^	12 ± 1	12 ± 1
	10^7^	10 ± 0	10 ± 0
***P. fluorescens***	10^6^	13 ± 1	11 ± 1
	10^7^	-	-

Note: Data expressed as means of three replicates ± standard deviations.

## Data Availability

The data presented in this study are available in article.
